# Temporäre postoperative Versorgung mit dem Knochenleitungshörsystem ADHEAR©: Eine prospektive Studie mit 76 Patienten

**DOI:** 10.1007/s00106-025-01548-w

**Published:** 2025-03-11

**Authors:** C. Dreyer, Z. Ahmad, Matthias Tisch, E. Goldberg-Bockhorn

**Affiliations:** 1 Klinik für Hals-Nasen-Ohrenheilkunde, Kopf-Hals-Chirurgie, Bundeswehrkrankenhaus Ulm, Oberer Eselsberg 40, 89081 Ulm, Deutschland; 2 Klinik für Hals-Nasen-Ohrenheilkunde, Kopf- und Halschirurgie, Universitätsklinikum Ulm, Ulm, Deutschland

**Keywords:** Knochenleitungshörgerät, Hörrehabilitation, Schallleitungsschwerhörigkeit, Ohrchirurgie, ADHEAR©, Bone conduction hearing aid, Hearing rehabilitation, Conductive hearing loss, Ear surgery, ADHEAR©

## Abstract

**Hintergrund:**

Im Rahmen von ohrchirurgischen Eingriffen kommt es durch die Tamponade zu einer vorübergehenden Schallleitungsschwerhörigkeit mit Einschränkung der Kommunikation. Diese kann durch eine unzureichende Hörverbesserung oder prolongierte Wundheilungsphase bis zum Folgeeingriff („second look“, Revision) fortbestehen.

**Ziel der Arbeit:**

Die vorliegende prospektive Studie untersucht den subjektiven und audiologischen Nutzen einer temporären Versorgung mit einem Knochenleitungshörgerät bei ohrchirurgisch versorgten Patienten.

**Material und Methoden:**

Für die Dauer der Tamponade wurden Patienten nach Ohrchirurgie mit dem Knochenleitungshörsystem ADHEAR© (Fa. MED-EL, Innsbruck, Österreich) versorgt. Anhand eines selbst entwickelten Fragebogens wurde die subjektive Zufriedenheit mit dem System erfasst. Neben den prä- und postoperativen Hörschwellen wurde nach Detamponade das Sprachverstehen in Ruhe mit und ohne Hörhilfe gemessen, um den Effekt der Versorgung auch nach der Detamponade bei Patienten mit anhaltender Schallleitungsschwerhörigkeit zu zeigen.

**Ergebnisse:**

Es wurden 76 Patienten getestet. 92 % der Patienten bewerteten die Hörhilfe während der Tamponade als nützlich. Mehr als 60 % stuften die Klangqualität als natürlich ein. 79 % bestätigten, das ADHEAR© bei einer erneuten Operation wieder tragen zu wollen. Nach Entfernung der Tamponade lag die Schallleitungskomponente bei 66 % der Patienten bereits bei < 10 dB. Durchschnittlich verbesserte sich das Sprachverstehen bei allen Patienten um 14 %, wobei die Patienten mit einer persistierenden Schallleitungskomponente ≥ 10 dB (*n* = 26) die größte Verbesserung um durchschnittlich 25 % erzielten.

**Schlussfolgerung:**

Die Ergebnisse der Studie zeigen den subjektiven und audiologischen Nutzen einer passageren Versorgung mit einem Klebehörgerät zur Überbrückung der Schallleitungsstörung nach Ohrchirurgie. Insbesondere bei persistierender Schallleitungsschwerhörigkeit könnte die Weiterversorgung mit dem ADHEAR© bis zur endgültigen Versorgung („second look“, Abschluss der Wundheilung, Revisionsoperation) Vorteile bieten.

**Zusatzmaterial online:**

Die Online-Version dieses Beitrags (10.1007/s00106-025-01548-w) enthält Tab. S1.

## Abstract (en)

**Background:**

The tamponade applied during ear surgery causes temporary conductive hearing loss with impairment of communication. This can persist until the follow-up procedure (second look, revision) due to insufficient hearing improvement or a prolonged wound healing phase.

**Objective:**

This prospective study investigates the subjective and audiological benefit of temporary fitting of a bone conduction hearing aid in patients undergoing ear surgery.

**Materials and methods:**

Patients were fitted with the bone conduction hearing system ADHEAR© (MED-EL, Innsbruck, Austria) after ear surgery for the duration of tamponade. A self-developed questionnaire recorded subjective satisfaction with the system. In addition to pre- and postoperative hearing thresholds, speech comprehension was measured in quiet with and without hearing aids after tamponade removal to show the effect of the treatment after tamponade removal in patients with persistent conductive hearing loss.

**Results:**

A total of 76 patients were tested and 92% rated the hearing aid as useful during the packed period. More than 60% rated the sound quality as natural and 79% confirmed that they would wear the ADHEAR© again if they had another ear surgery. After packing removal, the air–bone gap was below 10 dB in 66% of patients. On average, speech discrimination improved by 14% in all patients, whereby patients with a persistent sound conduction component ≥ 10 dB (*n* = 26) achieved the greatest improvement of 25% on average.

**Conclusion:**

The results of the study show subjective and audiological benefits with temporary fitting of an adhesive hearing aid to bridge the conductive hearing loss after ear surgery. Continued use of the ADHEAR© could benefit patients with persistent conductive hearing loss until the final fitting appointment (second look, completion of wound healing, revision surgery).

Schwerhörigkeit ist ein weit verbreitetes Problem, das rund 15 Mio. Menschen in Deutschland betrifft [1]. Je nach Art der Schwerhörigkeit kommen verschiedene Verfahren der Hörrehabilitation infrage, darunter konventionelle Hörgeräte, passive Mittelohrprothesen, aktive und passive Knochenleitungssysteme sowie aktive Mittelohrimplantate.

Nach ohrchirurgischen Eingriffen kann es zunächst durch die eingelegte Tamponade und später durch die noch nicht abgeschlossene Wundheilung oder eine noch unzureichende Hörverbesserung im Rahmen der Operation zu einer vorübergehenden Schallleitungsschwerhörigkeit kommen. In dieser Phase ist insbesondere bei kombinierter Schwerhörigkeit eine Versorgung mit einem konventionellen Hörgerät oft nicht sinnvoll, nicht wirtschaftlich oder technisch begrenzt.

Ein temporäres Knochenleitungshörgerät (KLH) wie das Klebehörgerät ADHEAR© (Fa. MED-EL, Innsbruck, Österreich) könnte jedoch eine sinnvolle Lösung sein. Das Funktionsprinzip des ADHEAR©-Systems ähnelt implantierbaren Knochenleitungshörsystemen und bietet den Vorteil einer einfachen und schnellen Anwendung mittels Klebeadapter, ohne aufwendigere Befestigungslösungen wie Softbänder oder Bügellösungen sowie ohne aufwendige Anpassung. Die Möglichkeit zeitlich begrenzter Leihverträge macht die Kostenübernahme durch die Kostenträger auch temporär möglich.

In einer Pilotstudie wurde bereits über eine signifikante Verbesserung des Sprachverstehens und eine hohe Patientenzufriedenheit mit dem ADHEAR©-System bei Patienten mit verschlossenem Gehörgang nach Mittelohroperation berichtet [2].

Ziel der vorliegenden prospektiven Studie war es, den subjektiven Nutzen dieser temporären Geräteversorgung in einer größeren Stichprobe zu untersuchen und zu prüfen, ob Patienten mit persistierender Schallleitungsschwerhörigkeit auch nach Entfernung der Tamponade weiterhin von der Geräteversorgung audiologisch profitieren. Zudem sollten Faktoren diskutiert werden, welche die temporäre Versorgung neben der Verbesserung der Lebensqualität rechtfertigen könnten.

## Material und Methoden

Bei der prospektiven Kohortenstudie im Vorher-Nachher-Design stellte jeder Patient seine eigene Kontrolle dar.

Die nachfolgenden Einschlusskriterien wurden angewendet:

Es wurden Patienten im Alter ab drei Jahren einbezogen, die nach ohrchirurgischen Eingriffen durch die Tamponade eine Schallleitungskomponente > 20 dB im Reintonaudiogramm über vier Frequenzen bei 0,5; 1; 2 und 4 kHz („pure tone average“, PTA4) aufwiesen. Als weitere Voraussetzung sollten die Patienten das ADHEAR©-System mit dem Klebeadapter tragen können. Vor dem Studieneinschluss musste die vom Patienten oder den Eltern (bei Minderjährigen) unterschriebene und datierte Informations- und Einwilligungserklärung vorliegen. Ausgeschlossen wurden Patienten, die Tests aufgrund von sprachlichen, körperlichen oder psychologischen Störungen nicht abschließen konnten.

**Abb. 1:**
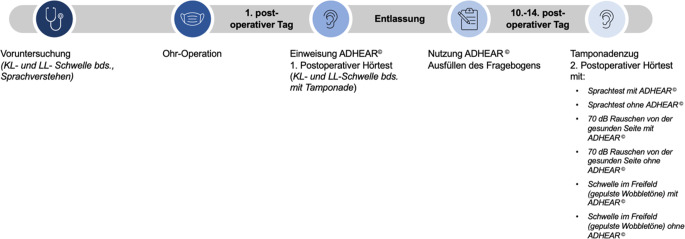
Studienablauf. *KL* Knochenleitung, *LL* Luftleitung

Studienteilnehmern wurde das ADHEAR© am Entlasstag nach der Operation hinter dem operierten Ohr aufgeklebt und die Handhabung erläutert. Die Indikationsgrenze des Knochenleitungshörgeräts von ≤ 25 dB HL (Hearing Level) in den Frequenzen 0,5 bis 4 kHz für das ADHEAR© wurde außer Acht gelassen, wenn Patienten einen subjektiven Nutzen durch das Klebehörgerät nach Erstanpassung angaben. Die Nachuntersuchung der Patienten erfolgte am Tag der Detamponade, 10 bis 14 Tage postoperativ (Abb. 1). Das KLH wurde nach der Freifeldmessung wieder abgegeben und aufbereitet, sodass es für weitere Studienpatienten zur Verfügung stand.

Für den Zeitraum mit Tamponade wurde die subjektive Zufriedenheit mit der Hörhilfe mittels eines hierfür entworfenen Fragebogens mit 13 Items vor der Tamponadenentfernung dokumentiert. Eltern füllten den Fragebogen hierbei für ihre Kinder aus. Der Fragebogen umfasste unter anderem Fragen zum ersten Höreindruck, dem subjektiven Nutzen, der Klangqualität, der Benutzungsdauer, der Verwendbarkeit für Brillenträger, der Tragedauer und der Hautverträglichkeit des Klebeadapters. Es handelte sich hierbei um einen nicht validierten Fragebogen.

Luft- und Knochenleitungsschwellen wurden prä- und postoperativ mit eingelegter Tamponade über Kopfhörer bei 0,25; 0,5; 1; 1,5; 2; 3; 4 und 6 kHz ermittelt und daraus jeweils der durchschnittliche Hörverlust der vier Hauptfrequenzen des PTA4 berechnet. Die Differenz aus dem durchschnittlichen Hörverlust in der Knochen- und Luftleitung ergab die durchschnittliche Schallleitungskomponente („air-bone gap“, ABG). Nach Entfernung der Tamponade wurden die Hörschwellen bei 0,5; 1; 2 und 4 kHz und das Sprachverstehen bei 65 dB in Ruhe im Freifeld mit und ohne Verwendung des ADHEAR© bestimmt und daraus die durchschnittliche Hörverbesserung im Vergleich zum Sprachverstehen vor der Operation berechnet. Das kontralaterale Ohr wurde hierbei im Freifeld mit einem Störgeräusch von 70 dB vertäubt. Für die Sprachaudiometrie wurde der Freiburger Sprachtest verwendet. Der Lautsprecher befand sich während der Freifeldmessung in einem Meter Entfernung auf Kopfhöhe vor dem Patienten. Primärer Zielparameter dieser Studie war eine hohe subjektive Zufriedenheit. Als Sekundärziel wurde die audiologische Verbesserung des Sprachverstehens von mindestens 10 % im Sprachverstehen in Ruhe mit dem ADHEAR© nach Detamponade festgelegt. Eine hohe subjektive Zufriedenheit wird erreicht, falls weniger als 20 % der Nutzer die Hörhilfe als „nicht nützlich“ empfinden und mit dem ersten Höreindruck „nicht zufrieden“ sind.

## Datenerhebung und Statistik

Hinsichtlich des Kollektivs wurden Daten zu Alter, Geschlecht und Indikation für den ohrchirurgischen Eingriff erhoben (s. elektronisches Zusatzmaterial online). Diese Daten wurden deskriptiv ausgewertet. Für nominalskalierte Variablen (Geschlecht, ohrchirurgischer Eingriff) wurden absolute und relative Häufigkeiten berechnet. Für metrische Merkmale (Alter) wurden Mittelwert mit Standardabweichung sowie Minimum und Maximum bestimmt.

Die Studiendaten wurden mithilfe des D’Agostino-&-Pearson- und des Shapiro-Wilk-Tests auf Normalverteilung geprüft. „Sidak’s multiple comparison test“ wurde zum Vergleich der Einzelwerte der Knochenleitungsschwellen prä- und postoperativ verwendet. Die durchschnittlichen Luftleitungsschwellen sowie das Einsilberverstehen prä- und postoperativ wurden mit dem Wilcoxon-Test verglichen. Minimum und Maximum wurden in eckigen Klammern angegeben [Min; Max]. Ein *p*-Wert von < 0,05 wurde als statistisch signifikant festgelegt. Die lineare Regression wurde verwendet, um den Zusammenhang zwischen der Schallleitungskomponente und der Verbesserung im Sprachverstehen durch das ADHEAR© zu untersuchen. Studiendaten wurden in Excel (Fa. Microsoft, Redmond, USA) erhoben. Die statistische Analyse und Erstellung der Graphen wurde mit GraphPad Prism 6 (Fa. GraphPad Software, San Diego, USA) durchgeführt.

Die Stichprobengröße basierte auf einer Studie von Weiss et al. [2], in der die Ergebnisse im Word Recognition Score (WRS) bei Patienten mit Tamponade ohne Hörhilfe 29 ± 20,0 % und mit Hörhilfe 75 ± 16,2 % betrugen. Für einen gepaarten t‑Test, ein Alpha von 0,05 und eine Power (1-Beta) von 95 % errechneten wir auf der Grundlage dieser Messungen eine Effektgröße von 2,5095 und eine minimale Stichprobengröße von 5. Um 20 % Studienabbrecher zu berücksichtigen, wurde die minimale Stichprobengröße mit 6 festgesetzt.

Die Studie wurde durch die Ethikkommission der Universität Ulm positiv bewertet (Ethikvotum 325/19).

## Ergebnisse

### Studienpopulation

Diese Studie rekrutierte im Zeitraum von 2019 bis 2021 insgesamt 76 Patienten. 39 davon waren männlich (51 %), 37 weiblich (49 %). Der Altersdurchschnitt betrug 38,7 Jahre (4 Jahre, 82 Jahre). Die Studienpopulation enthielt 9 Kinder unter 14 Jahren und 4 Jugendliche. Die Indikation zur Tympanoplastik wurde in 36 Fällen (47 %) wegen einer chronisch mesotympanalen Otitis media (CMO) und in 23 Fällen (30 %) wegen eines Cholesteatoms gestellt. Zwei Patienten erhielten eine Meatoplastik bei Gehörgangsexostosen (3 %). Bei 15 Patienten mit Otosklerose (20 %) wurde eine Stapesplastik durchgeführt. Ein Patient erhielt im Rahmen der Tympanoplastik ein aktives Mittelohrimplantat.

Lediglich ein Studienteilnehmer musste wegen einer Unverträglichkeit auf den Klebstoff die Studie vorzeitig beenden.

### Subjektive Patientenzufriedenheit

Bei Vorstellung zur Detamponade wurde die Handhabung und subjektive Zufriedenheit während der Zeit mit Tamponade anhand eines Fragebogens bei allen Teilnehmern erfasst. 89 % der Patienten waren bereits mit dem ersten Höreindruck mit dem ADHEAR© zufrieden. Am Studienende empfanden 92 % der Patienten die Hörhilfe als nützlich. 79 % gaben an, das Gerät bei einer erneuten Ohroperation wieder nutzen zu wollen. Die Lautstärke wurde von 82 % der Nutzer als ausreichend und die Klangqualität von 71 % als natürlich empfunden. Die Verwendung der Hörhilfe war bei 81 % problemlos. 19 % berichteten über Probleme, z. B. Pfeifen bei Berührung, Probleme beim Tragen einer Kopfbedeckung oder eines Fahrradhelms, aber auch Schwierigkeiten bei Benutzung des Klebeadapters oder des Audioprozessors.

**Abb. 2:**
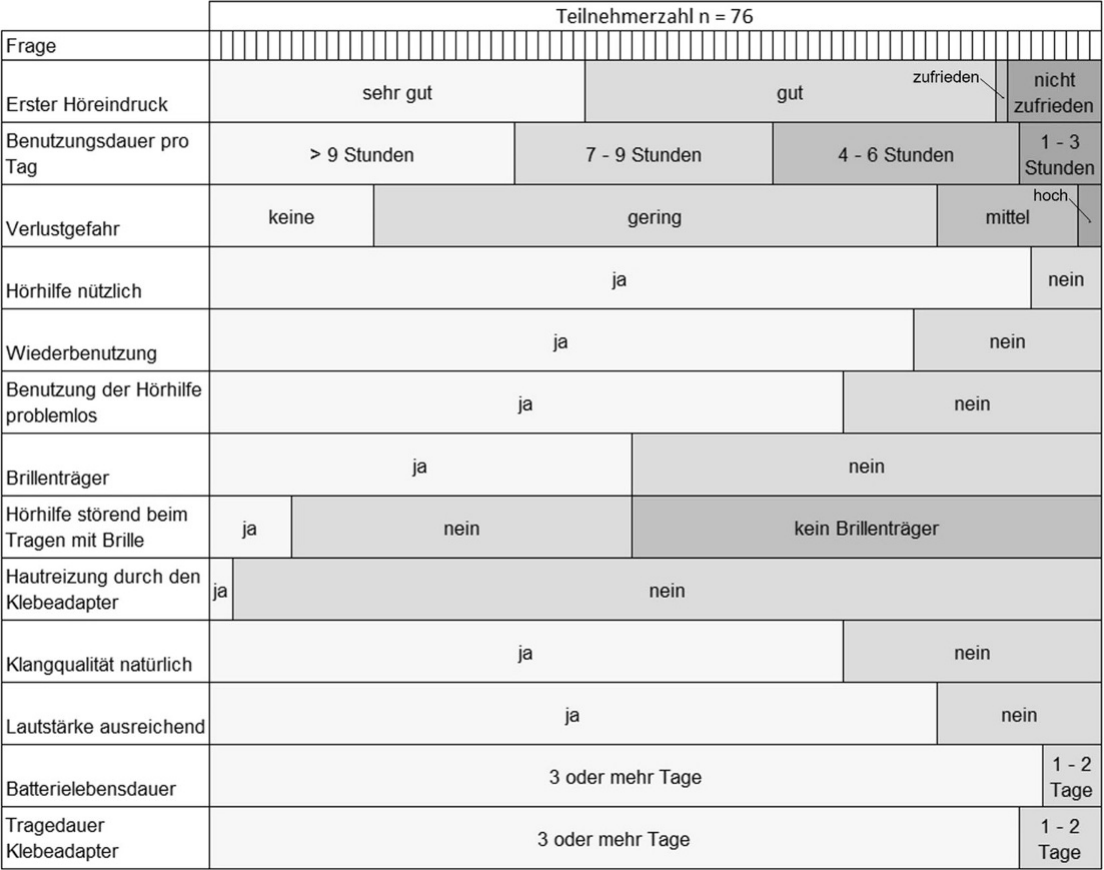
Fragebogenergebnisse nach Verwendung des ADHEAR©-Hörsystems während der Tamponade (*n* = 76)

47 % der Studienteilnehmer waren Brillenträger. Von diesen empfanden 19 % das KLH als störend bei gleichzeitigem Tragen der Brille. Eine Hautreaktion durch den verwendeten Klebstoff trat bei 3 % der Patienten auf. Die überwiegende Zahl der Nutzer (91 %) wechselte den Klebeadapter nach frühestens 3 Tagen. 9 % gaben an, die Hörhilfe nur 1 bis 3 h am Tag zu verwenden. 63 % verwendeten das Gerät 7 h oder mehr. Die Ergebnisse des Fragebogens sind in Abb. 2 zu sehen.

### Audiologische Ergebnisse

**Abb. 3:**
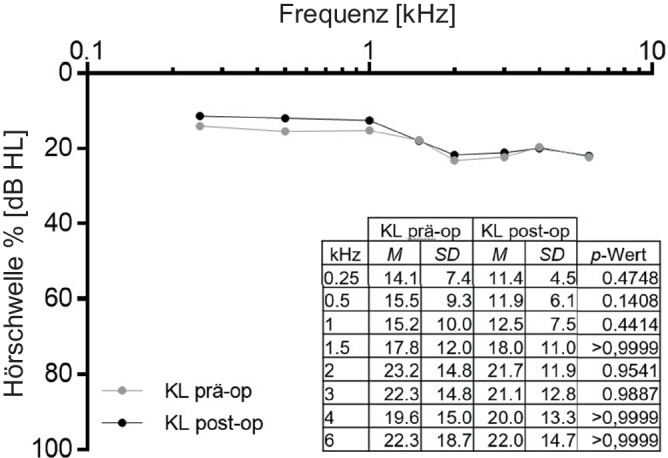
Mittelwerte der Knochenleitungsschwellen vor (*grau*) und nach (*schwarz*) der Operation. Die Messwerte zeigten in keiner Frequenz signifikante Unterschiede. *n* = 75, *KL* Knochenleitungsschwelle

Der durchschnittliche präoperative Hörverlust betrug im gesamten Kollektiv für die Knochenleitung 18 ± 10 dB und für die Luftleitung 35 ± 18 dB. Die Knochenleitungsschwellen blieben postoperativ stabil (PTA4_KL_ präop.: 18 ± 10 dB; PTA4_KL_ postop.: 17 ± 9 dB, Abb. 3).

**Abb. 4:**
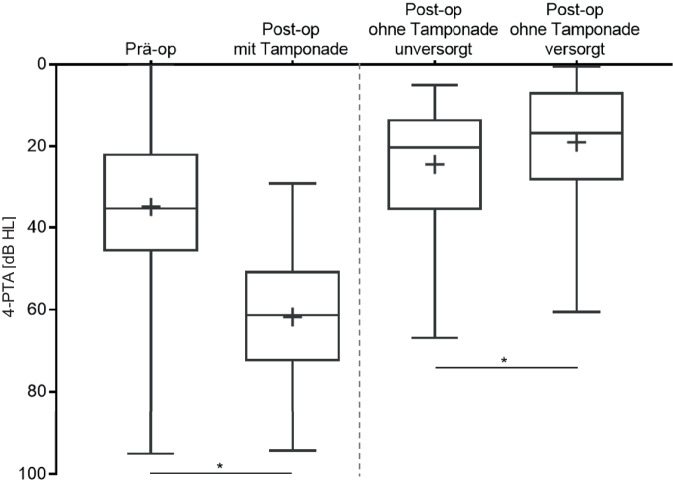
Durchschnittliche Hörschwelle (PTA4) prä- und postoperativ

Nach Operation und Einbringen der Tamponade verschlechterten sich die mit Kopfhörern gemessenen Luftleitungsschwellen statistisch signifikant (*p* < 0,0001) auf durchschnittlich 62 ± 14 dB. Die durchschnittliche Schallleitungskomponente verschlechterte sich von 17 ± 15 dB präoperativ auf 45 ± 13 dB postoperativ unter liegender Tamponade. Nach Entfernung der Tamponade wurde im Freifeld ein mittlerer Hörverlust (PTA4) von 24 ± 15 dB gemessen. Mit dem KLH konnte ohne eingelegte Tamponade eine signifikante Verbesserung der Hörschwelle auf durchschnittlich 19 ± 14 dB erzielt werden (*p* < 0,0001; Abb. 4).

Der durchschnittliche Hörverlust nahm bezogen auf die Luftleitungsschwellen durch die Tamponade signifikant zu (*p* < 0,0001). Das ADHEAR© verbesserte den durchschnittlichen Hörverlust im Freifeld nach Entfernung der Tamponade signifikant (*p* < 0,0001). *n* = 76.

Nach Detamponade betrug der ABG im Mittel 8,0 ± 11 dB (5 dB; 44 dB).

**Abb. 5:**
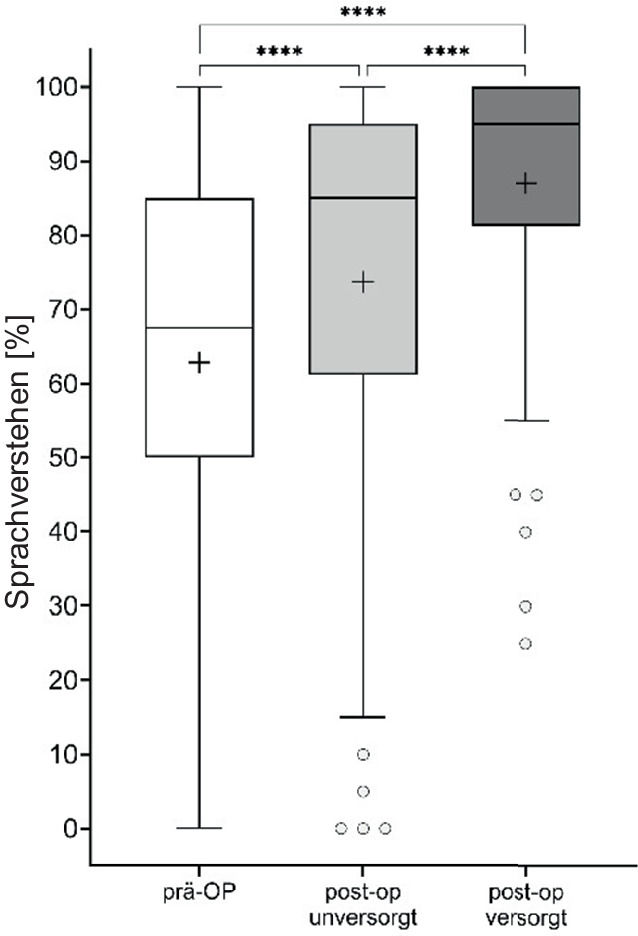
Prä- und postoperatives Sprachverstehen nach Detamponade unversorgt und mit ADHEAR© versorgt (*p* < 0,0001)

Das Sprachverstehen in Ruhe bei 65 dB SPL (Sound Pressure Level) wurde zu zwei verschiedenen Zeitpunkten, nämlich präoperativ und postoperativ bei Entfernung der Tamponade, gemessen. Bei der Vorstellung zur Detamponade wurde das Sprachverstehen unversorgt und mit KLH versorgt ermittelt. Die Abb. 5 zeigt die Ergebnisse der Sprachtests von allen Patienten prä- und postoperativ bzw. unversorgt und mit KLH versorgt.

Das präoperative Sprachverstehen bei 65 dB SPL wurde im Durchschnitt von 65 ± 25 % durch den operativen Eingriff auf 74 ± 28 % verbessert, welches einen Operationseffekt von etwa 10 % Verbesserung im Durchschnitt zeigt. Jedoch konnte durch die Benutzung des KLH ein Sprachverstehen von 87 ± 18 % erreicht werden. Alle Unterschiede erwiesen sich statistisch als hochsignifikant (*p* < 0,0001).

**Abb. 6:**
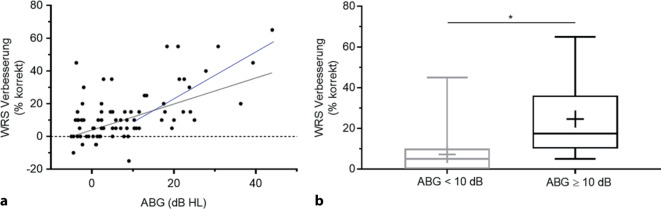
Verbesserung des Einsilberverstehens in Ruhe durch das ADHEAR© in Abhängigkeit von der Schallleitungskomponente. **a** Die Datenpunkte repräsentieren individuelle Patientenergebnisse. Die *schwarze* Regressionsgerade (y = 3,95 + 0,796 × x) wurde für alle 76 Datenpunkte berechnet. Die *blaue* Regressionsgerade (y = −5,14 + 1,397 × x) wurde für Patienten bestimmt, welche ein ABG von ≥ 10 dB aufwiesen (*n* = 26). **b** Patienten mit einem ABG ≥ 10 dB profitierten signifikant mehr von der ADHEAR©-Versorgung nach Detamponade als Patienten mit geringerem ABG (*p* > 0,0001). *ABG* „air-bone gap“

Die Ausprägung des postoperativen ABG korrelierte positiv mit der Verbesserung des Sprachverstehens durch das ADHEAR© (Abb. 6a). Patienten mit einem ABG ≥ 10 dB (*n* = 26) profitierten dabei noch stärker von der ADHEAR©-Versorgung (Abb. 6b). In dieser Gruppe betrug der durchschnittliche ABG 21 ± 9,2 dB. Das Einsilberverstehen bei 65 dB verbesserte sich bei Patienten mit ABG ≥ 10 dB (*n* = 26) von 56 % auf 81 %, wohingegen Patienten mit ABG < 10 dB (*n* = 50) ihr Sprachverstehen durchschnittlich von 83 % auf 90 % verbesserten (Abb. 6).

## Diskussion

Der audiologische Nutzen eines KLH für die Behandlung von Schallleitungsschwerhörigkeiten wurde bisher in mehr als 130 Studien untersucht und bestätigt. In Studien bei Erwachsenen ergab sich ein mittlerer funktionaler Gewinn (MFG), dem Vergleich von unversorgter und versorgter Freifeldschwelle, von 15 bis 25 dB HL [2–9]. Waren die Studienteilnehmer Kinder, zeigte sich ein höherer MFG von 23 bis 36 dB HL [10–13]. Der erzielte Gewinn im Wortverstehen in Ruhe mit dem KLH betrug 17 bis 57 % bei Erwachsenen und 55 bis 76 % bei Kindern [3–5, 7, 9]. Die Unterschiede der audiologischen Ergebnisse zwischen den Studiengruppen begründen sich dabei allerdings in den zugrunde liegenden Pathologien und nicht im Alter. Während in den Kinderstudien gut zu versorgende Atresien mit reiner Schallleitungsschwerhörigkeit die Ursache waren, lagen bei den Erwachsenenstudien vor allem Cholesteatome als Grund für die Schwerhörigkeit vor, die aufgrund der kombinierten Schwerhörigkeit deutlich schlechter zu versorgen waren. In unserer eigenen Untersuchung, die neun Kinder unter 14 Jahren und fünf Jugendliche (unter 18 Jahren) beinhaltete, konnte die hohe subjektive Zufriedenheit unabhängig von der Operationsindikation und dem durchgeführten chirurgischen Eingriff nachdrücklich bestätigt werden. Im Fragebogen gaben die Eltern der Kinder und Jugendlichen an, dass es insbesondere zu einer Verbesserung der Diskrimination im Schulunterricht kam. Auch wurde die verbesserte Verständlichkeit in der Freizeit als Hauptargument für das Tragen des ADHEAR© genannt.

Ob die Leistungsfähigkeit eines Klebehörgeräts ausreichend ist, eine entsprechende Verstärkungsleistung bei gleichzeitig guter Klangqualität zu erbringen, wurde zwischenzeitlich ebenfalls umfassend untersucht. Mehrere Studien konnten zeigen, dass das Klebehörgerät eine äquivalente audiologische Leistung im Vergleich zu konventionellen Knochenleitungsgeräten am Stirnband erbringt [7, 10, 14]. Pedrero et al. [12] konnten zeigen, dass das Klebehörgerät außerdem vergleichbare Verbesserungen wie passive transkutane Knochenleitungsimplantate erzielt.

Allen Studien gemeinsam ist die hohe Akzeptanz und subjektive Zufriedenheit der Patienten mit dieser Hörhilfe [10, 15], selbst über einen Zeitraum von einem Jahr und mehr [16]. In einer Untersuchung von Weiss et al. [2] wurde erstmals der Nutzen des ADHEAR© als passagere Hörhilfe nach Ohroperation untersucht. Hierbei zeigte sich eine gute Hörverstärkung bei hoher Akzeptanz und Zufriedenheit der untersuchten Patienten. Ob dies auch für ein großes Patientenkollektiv – unabhängig vom Alter – gilt, war bislang nicht untersucht worden. Um möglichst realitätsnahe Verhältnisse widerzuspiegeln, entschieden wir uns daher in der vorliegenden Untersuchung dafür, Patienten unabhängig vom Alter und der Art der durchgeführten Ohroperation für die Zeitdauer der postoperativen Tamponade und teilweise deutlich darüber hinaus (auf ausdrücklichem Patientenwunsch) mit einem Klebehörgerät zu versorgen und zu untersuchen, ob hierdurch ein fassbarer Vorteil für die Patienten resultiert. Es handelt sich hierbei um die erste und bislang größte Untersuchung zur subjektiven Effektivität eines Klebehörgeräts. Studienergebnisse nach Cholesteatomchirurgie zeigten, dass ein relevanter Anteil an Patienten eine konventionelle Hörgeräteversorgung aus verschiedenen Gründen trotz potenzieller Hörverbesserung postoperativ ablehnt oder aufgrund unterschiedlicher Probleme nicht tragen kann [17]. Die langfristige Akzeptanz des ADHEAR©, insbesondere bei einseitiger Schwerhörigkeit, und ein eventuell bestehender Vorteil gegenüber einer konventionellen Hörgeräteversorgung sollte daher in weiteren Studien untersucht werden.

Die Knochenleitungsschwellen einzelner Patienten lagen nicht im Indikationsbereich des ADHEAR©. Die Patienten wurden jedoch aufgrund des subjektiven Benefits bei Erstanpassung trotzdem versorgt und in die Studie eingeschlossen. Der subjektive Benefit kann hier möglicherweise auch durch die Stimulation des normalhörenden Gegenohrs erklärt werden. Eine geringe Anzahl von Patienten (*n* = 4) befand sich beidohrig außerhalb der Geräteindikation. Dass solche Patienten dennoch von einer Versorgung mit dem ADHEAR© profitieren können, konnte bereits in einer Untersuchung von Dahm et al. [6] gezeigt werden.

Im Vergleich der prä- und postoperativen Knochenleitungsschwellen zeigte sich keine signifikante Veränderung, was für die Qualität der durchgeführten Chirurgie spricht.

Durch die Tamponade ergab sich innerhalb unserer Studienpopulation eine durchschnittliche Luftleitungsschwelle von 62 dB für das tamponierte Ohr, was einem mittel- bis hochgradigen Hörverlust entspricht. Zum Vergleich zeigte sich in der Untersuchung von Weiss et al. [2] eine durchschnittliche Hörschwelle von 43 dB. In einer Untersuchung an 10 Patienten konnte ein MFG von durchschnittlich 15 bis 19 dB bei Verwendung des KLH während der Zeit der Tamponade nachgewiesen werden. Unsere Ergebnisse aus der Fragebogenerhebung zur Patientenzufriedenheit untermauern die bei Weiss et al. [2] nachgewiesene Effektivität der Versorgung mit einem ADHEAR© während der Tamponade anhand eines vergleichsweise großen Kollektivs. 88 % unserer Patienten gaben an, mit dem ersten Höreindruck bei Umgebungslärm sehr zufrieden oder zufrieden gewesen zu sein. Dieses Ergebnis ist deutlich besser als vergleichbare Ergebnisse mit konventionellen Hörgeräten. Eine große Umfrage der BARMER Krankenkasse aus 2019 ergab eine Zufriedenheit von 72 % („zufrieden“ und „sehr zufrieden“ kumuliert) [18].

In der Untersuchung von Weiss et al. [2] wurde die Phase bis zur Detamponade untersucht. Unsere Arbeit konnte nun zusätzlich aufzeigen, dass eine weiterführende Versorgung mit dem Knochenleitungshörgerät gerade für solche Patienten effektiv sein kann, welche nach Entfernung der Tamponade noch unter einer persistierenden Schallleitungsschwerhörigkeit von mehr als 10 dB leiden. Hier ergibt sich insbesondere für Patienten mit stark sezernierenden Radikalhöhlen oder nicht möglicher Hörverbesserung im ersten Schritt eine gute Möglichkeit einer passageren Hörrehabilitation. Die Korrelation zeigt hierbei, dass der zu erwartende Hörgewinn mit zunehmender Schallleitungskomponente ansteigt. Dieses Ergebnis ist signifikant, ebenso wie der Hörgewinn nach Detamponade durch die durchgeführte Ohroperation signifikant war. Trotz der signifikanten Verbesserung der Einsilberverständlichkeit durch die Ohroperation (Abb. 5) konnte die Einsilberverständlichkeit durch das Klebehörgerät nochmals signifikant gesteigert werden (Abb. 5).

Dass das Klebehörgerät auch über eine längere Zeit zuverlässig als passagere Hörhilfe nutzbar ist, zeigen die Ergebnisse des Fragebogens. Fast zwei Drittel unserer Patienten trugen das Hörgerät mehr als 7 h pro Tag. Dies stellt im Vergleich zu konventionellen Hörgeräten – trotz des Aufwands des Klebeadapters – einen vergleichsweise guten Wert dar. In der aktuellen EuroTrak-Studie konnte für konventionelle Hörgeräte eine durchschnittliche Tragedauer von 8,9 h/Tag erhoben werden [19].

Mehr als 75 % der Studienteilnehmer waren sowohl mit der Verstärkungsleistung als auch mit der empfundenen Lautstärke zufrieden. Diese Ergebnisse stehen im Einklang mit den zahlreichen Publikationen, welche den audiologischen Nutzen des ADHEAR© und eine hohe subjektive Zufriedenheit bei anderen Indikationen als der hier untersuchten bestätigen [10, 15, 16]. Diese Akzeptanz bekommt umso mehr Gewicht, da 90 % der Studienteilnehmer vor der Studie noch nie ein Hörgerät getragen hatten, da bei einem Teil der Studienteilnehmer noch keine Indikation bestand und andere wiederum schlichtweg noch nicht mit einem Hörgerät versorgt waren. Es handelte sich also um ein Patientenkollektiv, das gewohnt war, beidohrig einen natürlichen, nicht verstärkten Klang zu empfinden. Die Tatsache, dass durch das KLH dieses Gefühl nicht oder nur wenig eingeschränkt wurde, zeigt, dass insbesondere bei annähernd normaler Knochenleitungshörschwelle KLH und insbesondere das ADHEAR© in der Lage sind, einen natürlichen Höreindruck zu erzeugen.

Wesentliche Probleme mit dem Klebeadapter traten nicht auf. Das Handling wurde als überwiegend problemlos beschrieben. Im Schnitt wurde der Klebeadapter alle 3–4 Tage gewechselt. Dies ist kongruent zu anderen publizierten Untersuchungen [2], die vergleichbare Tragedauern beschrieben. Lediglich ein Studienteilnehmer musste wegen einer Unverträglichkeit auf den Klebstoff die Studie vorzeitig beenden.

Als weitere mögliche Limitation der Studie ist der fehlende Vergleich mit anderen nichtinvasiven KLH (z. B. KLH am Stirnband) als Alternative hinsichtlich des Tragekomforts zu nennen. Das Alter der Probanden kann Einfluss auf die Verlässlichkeit von Sprachtestergebnissen bei Kindern und älteren Personen hinsichtlich Konzentration und Erschöpfung nehmen, was einschränkend bei der Betrachtung der vorliegenden Studienergebnisse bedacht werden muss. Sprachtests im Störgeräusch und der Einfluss des kontralateralen Ohrs auf die Hörergebnisse und die Patientenzufriedenheit sollten in zukünftigen Untersuchungen berücksichtigt werden. Die Aussagekraft der Fragebogenanalyse ist aufgrund der fehlenden Validierung eingeschränkt. Um die allgemeine Gültigkeit und die Messgenauigkeit des entwickelten Fragebogens nachzuweisen, sollte in nachfolgenden Studien eine Validierung vorgenommen werden.

## Ausblick

Die vorliegende prospektive Studie konnte in einer vergleichsweise großen Stichprobe von 76 Patienten den subjektiven und audiologischen Nutzen einer Versorgung zur Überbrückung eines zeitlich begrenzten Hörverlusts mithilfe des ADHEAR© Hörgeräts aufzeigen, die sich mit den Erkenntnissen von Weiss et al. (2019) decken.

Die Ergebnisse zeigten dabei folgende Vorteile:

- Schnelle, einfache und sichere Versorgung ohne aufwendige Anpassung
- Hohe Akzeptanz bei allen Patientengruppen
- Besondere Eignung für Patienten, bei denen absehbar ist, dass postoperativ eine relevante Schallleitungskomponente bestehen wird und eine Versorgung mit konventionellen Hörgeräten nicht möglich ist oder langfristig nicht erfolgen soll, wie z. B. bei geplantem „second look“ ohne initialen Höraufbau, Wundheilungsstörung oder stark sezernierenden Radikalhöhlen

Allerdings müssen auch einige Nachteile berücksichtigt werden, darunter:

- Kosten und Finanzierung sind derzeit noch nicht geklärt, da die Kostenübernahme durch die Krankenkasse nicht garantiert ist
- Organisatorische Aspekte wie die Verantwortlichkeit der Ausgabe und Erläuterung der Verwendung
- Die Rentabilität kann bei sehr kurzer Nutzungsdauer z. B. während der Tamponade fraglich sein

## Fazit für die Praxis

- Zusammenfassend lässt sich sagen, dass sich das ADHEAR© für Patienten nach Ohroperationen, bei denen aus verschiedenen Gründen passager postoperativ eine versorgbare Schwerhörigkeit besteht, eignet.
- Als kurz- und mittelfristige Lösung vor dem Hintergrund der aktuellen Problematik einer verzögerten Chirurgie bei Kindern mit chronischem Paukenerguss und Adenoiden können sich darüber hinaus zusätzliche sinnvolle Indikationen ergeben, die einer Bewertung seitens der Kostenträger bedürfen.
- Es ist jedoch zu beachten, dass weitere Untersuchungen und Klärungen bezüglich der Kosten, Finanzierung und organisatorischen Aspekte erforderlich sind.

## Supplementary Information

Tab. S1 Demografische Daten der Studienteilnehmer

## Data Availability

Die erhobenen Datensätze können auf begründete Anfrage in anonymisierter Form beim korrespondierenden Autor angefordert werden.
