# Supplementary material for: Temporary postoperative care with the bone conduction hearing system ADHEAR©: A prospective study on 76 patients
Source: HNO. 2025 Mar 11;73(4):233–41. [Article in German] doi: 10.1007/s00106-025-01548-w (PMC11926046; doi:10.1007/s00106-025-01548-w)
Supplement: Supplementary file 1 — Tab. S1 Demografische Daten der Studienteilnehmer [file 106_2025_1548_MOESM1_ESM.pdf]

Tab. S1: Demografische Daten der Studienteilnehmer

Pat. Nr.	Alter	Geschlecht	Diagnose	OP Typ	KL PTA4, kontra-lateral (dB HL)	LL PTA4, kontra-lateral (dB HL)	KL PTA4, ipsi-lateral (dB HL)	LL PTA4, ipsi-lateral (dB HL)
1	48	weiblich	COM	Tymp IV	14	19	28	43
2	33	männlich	Cholestatom	Tymp IIIc	16	35	24	48
3	40	männlich	COM	Tymp IIIb	14	15	19	28
4	53	männlich	Otosklerose	Stapesplastik	36	50	50	76
5	72	weiblich	Otosklerose	Tymp I	20	20	33	41
6	45	männlich	Otosklerose	Stapesplastik	4	4	13	36
7	22	männlich	COM	Tymp IIIc	1	0	10	31
8	17	weiblich	Otosklerose	Stapesplastik	13	15	20	38
9	45	männlich	COM	Tymp I	18	19	20	28
10	33	weiblich	Otosklerose	Stapesplastik	10	18	11	31
11	8	weiblich	Cholestatom	Tymp I	7	13	6	22
12	62	männlich	COM	Tymp I	29	31	26	48
13	8	männlich	COM	Tymp I	5	1	5	7
14	35	weiblich	COM	Tymp IIIc	12	14	12	20
15	47	weiblich	COM	Tymp I	11	6	13	13
16	32	männlich	COM	Tymp IIIc	6	4	n. a.	95
17	13	weiblich	Cholestatom	Tymp I	3	0	7	35
18	23	weiblich	COM	Tymp IIIb	4	0	6	50
19	41	männlich	Otosklerose	Stapesplastik	13	25	13	25
20	67	weiblich	Cholestatom	Tymp I	13	9	31	35
21	63	männlich	Cholestatom	Tymp IIIc	10	12	14	36
22	23	weiblich	COM	Tymp I	5	2	8	47
23	6	männlich	Cholestatom	Tymp IIIc	12	10	23	48
24	70	weiblich	Gehörgangs-exostose	Gehörgangs-plastik	47	46	41	46
25	53	weiblich	Cholestatom	Tymp IIIc	9	5	22	71

26	52	männlich	Otosklerose	Stapesplastik	23	36	25	49
27	50	weiblich	COM	Tymp IIIc	7	6	15	31
28	39	männlich	Otosklerose	Stapesplastik	16	28	33	45
29	50	männlich	Otosklerose	Stapesplastik	32	53	43	63
30	55	weiblich	COM	Tymp IIIc	44	40	40	83
31	45	weiblich	Cholestatom	Tymp IIIc	5	4	6	13
32	24	männlich	Cholestatom	Tymp IIIb	3	0	12	26
33	30	männlich	COM	Tymp I	26	32	5	3
34	54	weiblich	COM	Tymp I	8	7	16	45
35	35	weiblich	Otosklerose	Stapesplastik	3	0	14	21
36	59	weiblich	COM	Tymp IIIb	5	1	11	39
37	40	männlich	Otosklerose	Stapesplastik	15	36	27	51
38	41	männlich	Otosklerose	Stapesplastik	21	20	21	41
39	60	weiblich	Cholestatom	Tymp IIIb	24	35	19	38
40	48	weiblich	Otosklerose	Stapesplastik	11	10	30	42
41	14	männlich	Cholestatom	Tymp IIIc	5	1	17	37
42	51	weiblich	Cholestatom	Tymp IIIc	10	7	24	41
43	55	männlich	COM	Tymp IIIc	14	25	17	41
44	62	männlich	COM	Tymp I	25	23	35	38
45	11	weiblich	Cholestatom	Tymp I	11	8	12	19
46	63	weiblich	COM	Tymp I	6	3	14	24
47	66	weiblich	Cholestatom	Tymp IIIb	35	51	21	35
48	22	weiblich	Otosklerose	Stapesplastik	11	14	9	15
49	46	männlich	COM	Tymp I	8	5	11	13
50	58	männlich	COM	Tymp I	7	6	11	10
51	11	männlich	Cholestatom	Tymp IIIc	6	3	6	31
52	46	weiblich	Cholestatom	Tymp IIIc	8	8	16	45
53	32	weiblich	Otosklerose	Stapesplastik	14	21	16	28
54	22	weiblich	COM	Tymp IIIc	7	5	17	31
55	21	männlich	COM	Tymp I	4	0	4	0
56	49	männlich	COM	Tymp I	13	10	20	23

57	60	weiblich	COM	Tymp IIIb	19	44	22	31
58	40	weiblich	COM	Tymp IIIc	7	4	17	21
59	9	männlich	Gehörgangs-exostose	Gehörgangs-plastik	4	0	4	0
60	33	männlich	COM	Tymp I	5	2	8	13
61	38	männlich	COM	Tymp IIIc	10	7	20	49
62	38	weiblich	COM	Tymp I	5	2	16	24
63	17	männlich	Cholestatom	Tymp IIIc	5	1	13	37
64	20	männlich	COM	Tymp IIIc	3	1	21	59
65	40	weiblich	COM	Tymp I	18	15	17	24
66	45	männlich	Cholestatom	Tymp I	4	1	9	8
67	18	männlich	Cholestatom	Tymp IIIc	5	1	13	33
68	34	weiblich	Otosklerose	Stapesplastik	10	8	28	44
69	30	männlich	Cholestatom	Tymp IV	4	0	11	16
70	8	weiblich	COM	Tymp I	6	0	16	53
71	36	weiblich	Cholestatom	Tymp IIIc	6	0	12	19
72	75	männlich	Cholestatom	Vibrant MedEL	31	25	37	43
73	82	männlich	COM	Tymp I	21	15	25	19
74	26	männlich	Cholestatom	Tymp IIIc	6	0	14	39
75	4	männlich	COM	Tymp I	6	0	16	53
76	17	weiblich	COM	Tymp I	6	0	45	53

nicht auswertbar (n. a.)
